# Lemongrass extract enhances productive performance, blood biomarkers, immunity, and gut health of broilers

**DOI:** 10.1016/j.heliyon.2024.e37783

**Published:** 2024-09-11

**Authors:** Amany A. El-Sahn, Eman A. Manaa, Amal M. EL-Barbary, Ayman M. Khalifah, Sahar Fayez, Asmaa S.A. Abdel-Daim, Ghadeer M. Albadrani, Mohamed M. Abdel-Daim, Mervat A. Abdel-Latif

**Affiliations:** a Poultry Breeding Research Department, Animal Production Research Institute, Agriculture Research Center, Giza, Egypt; b Animal and Poultry Production, Department of Animal Wealth Development, Faculty of Veterinary Medicine, Benha University, Toukh, 13736, Egypt; c Livestock Research Department, Arid lands Cultivation Research Institute, City of Scientific Research and Technological Applications (SRTA – City), New Borg EL Arab, Egypt; d Department of Histology and Cytology, Faculty of Veterinary Medicine, Damanhour University, Damanhour, 22511, Egypt; e Department of Nutrition and Clinical Nutrition, Faculty of Veterinary Medicine, Beni-Suef University, Beni-Suef, 62511, Egypt; f Department of Biology, College of Science, Princess Nourah bint Abdulrahman University, Riyadh, Saudi Arabia; g Department of Pharmaceutical Sciences, Pharmacy Program, Batterjee Medical College, Jeddah, Saudi Arabia; h Pharmacology Department, Faculty of Veterinary Medicine, Suez Canal University, Ismailia, Egypt; i Department of Nutrition and Veterinary Clinical Nutrition, Faculty of Veterinary Medicine, Damanhour University, Damanhour, 22511, Egypt

**Keywords:** Broiler, Gene expression, Growth, Histology, Lemongrass

## Abstract

**Background:**

Lemongrass (LG) had various phytochemical components such as saponins, phenols, resins, alkaloids, tannins, flavonoids, glycosides and terpenes, minerals as well as vitamin C which had various pharmacological actions (antioxidant, anti-inflammatory, antibacterial, antibiotic, and antifungal) and growth promoter. The use of LG in broiler nutrition can be optimized the bird performance and gut health. Based on the high nutrition value of LG and absence of sufficient studies on the effect of lemongrass aqueous extract (LGX) on broiler performance and gut health (antioxidant and immune biomarkers and intestinal morphology), the aim of the present study is to investigate the impact of using LGX on productive performance, blood biomarkers, immunity and gut health of broilers.

**Methods:**

A total of two hundreds one-day- old male broiler chicks (Cobb 500) were fed on the starter basal diet for 6 days. From day 7 of age onwards, the birds were distributed, at random, into 4 groups. Each group included 5 replicates with 10 chicks per replicate. The birds in group 1 were not administered lemongrass extract (control, LGX0) while chicks in group 2 (LGX100), 3 (LGX200) and 4 (LGX300) were administered the aqueous extract of lemongrass in drinking water at levels of 100, 200 and 300 ml/l, respectively. The experimental period lasted for 35 day. Growth performance parameters, economic efficiency, hematological and biochemical biomarkers, expression of some antioxidant and immune related genes, cecal bacterial counts and intestinal morphological changes all were assessed.

**Results:**

The results indicated that, administration of LGX in drinking water at levels of 200, 100 ml/l, respectively significantly improved (p ≤ 0.001) body weight (BW), body weight gain (BWG) and feed conversion ratio (FCR) than the control but without any effect on economic efficiency index (EEI) and feed intake (FI). On the other hand, the addition of LGX in drinking water at levels of 300 ml/l significantly decreased (p ≤ 0.001) FI, EEI and all growth performance parameters as compared to those other groups. LGX supplemented birds groups exhibited higher Hb, PCV, MCH, platelets, and lymphocytes than the control group. However, the ratio of H/L in LGX100 and LGX200 groups was lower (*p* ≤ 0.001) than other groups. LGX supplemented groups showed low (*p* ≤ 0.001) cholesterol, creatinine, MDA and high (*p* ≤ 0.01) TAC. Up regulation (*p* ≤ 0.001) of the expressions of catalase, GPX1, and SOD1 were in LGX200 group compared to other groups. While, the proinflammatory genes expression (IL1B, IL6, IFNᵧ, and TNF) were down regulated (*p* ≤ 0.001) in the LGX200 compared to others. Moreover, LGX200 and LGX300 reduced (*p* ≤ 0.001) the intestinal pathogens counts (*E.coli* and Salmonella). Administration of LGX at levels of 200 and 100 ml/l, respectively enhanced (*p* ≤ 0.001) villi height and crypts depth.

**Conclusions:**

It was concluded that lemongrass aqueous extract can be included at level 100 and 200 ml/l in broilersˈ drinking water since it resulted in improved weight, feed conversion ratio, blood parameters, immunity and gut health without any deleterious effect on the health and performance of the birds. LGX at a 200 ml/l supplementation level achieved the best results followed by a 100 ml/l level. Also, the tested supplements can be used as natural growth promoter instead of antibiotic and help in solving the global problem of antimicrobial resistant bacterial strains responsible for human and animal diseases.

## 1 Introduction

The growth of antimicrobial resistant bacterial strains responsible for human and animal diseases has led to global awareness of the need to use nature-derived antimicrobials for therapeutic and prophylactic purposes, as well as to enhance livestock performance [1]. The phytobiotics supplementation is an essential key to achieve this task [[2], [3], [4]]. Organic acids (formic, citric, acetic, butyric, etc.) are distinguished among others with its potency by adjusting the intestinal pH, improving digestion and nutrient absorption [5,6], and enhancing immune responses of broilers [7]. Many researches have focused on the application of citric acid that derived from different sources of fruits and herbs as poultry feed supplements. One of these herbs is Lemongrass (*Cymbopogon citrates*) which is an aromatic perennial grass (culinary herb). Many cultures have utilized it as a folk medicine, and it is grown in tropical and semi-tropical [8]. The most important constituent of LG is volatile oil which composed mainly of Citral (lemonal). Citral has various pharmacological actions (antioxidant, anti-inflammatory, antibacterial, antibiotic, and antifungal) and growth promoter [9,10]. In addition, lemongrass has other phytochemical components such as saponins, phenols, resins, alkaloids, tannins, flavonoids, glycosides and terpenes which protect the cells of the organism from oxidative stress by neutralizing and curbing harmful free radicals. These compounds are also distinguished by a biological efficacy that allows them to improve growth and health of bird's digestive system [11]. Lemongrass is rich in macro and trace elements [12], as well as vitamin C [13]. Citral, myrcene, and geraniol of LG can enhance the functioning of the immune system as it possesses antioxidant properties and thus improve the physiological characteristics and biochemical blood tests of broiler chickens [14]. The use of LG in broiler feeding led to a significant increase in total protein in broiler chicken serum, as well as a decrease in the activities of liver enzymes (AST) This may be due to bioactive compounds of LG that repair liver tissue or restore cellular permeability that can be caused by cytotoxic compounds [15]. It also indicated that LG can be significantly reduce the cholesterol level in the blood of birds due to its content of effective compounds that inhibit the activity of reductase (hydroxy-3- methylglutaryl-coenzyme A 3), which acts as an essential regulatory enzyme in the process of cholesterol synthesis [16]. Moreover, LG has been found to have an antibacterial effect against strains of *E. coli, S. aureus, and S. enterica* [11,17].

The high cost of poultry feeds motivates the researchers to use unconventional natural growth promoters such as LG herbal plant which is safe, cheap and maintain the optimum growth of birds by enhancement of feed utilization and gut health condition [18]. Lemon grass can be used as a promising alternative to antibiotics [6,19]. Several researchers have studied the substantial improvement in broiler performance and carcass characteristics of dietary herbal inclusion such essential citrus oils [20]. Moreover, citrus oils may increase thyroid hormone [21]. Also, lemongrass has antihypertensive effect in animals due to stimulation of parasympathetic activity [22]. However, their effects on the gut health regarding antioxidants and immune biomarkers, intestinal bacterial count and intestinal morphology weren't sufficiently studied. Therefore, this experiment was carried out to evaluate the impact of aqueous lemongrass extract inclusion in broiler drinking water and its effect on growth performance, economic efficiency, blood biomarkers (haematological and biochemical), antioxidants (serum TAC and MDA and expression of catalase, GPX1, and SOD1), immunity (expression of IL1B, IL6, IFNᵧ, and TNF) and gut health (intestinal bacterial count and intestinal morphology).

## 2 Materials and methods

### 2.1 Dietary treatments and management

A total of two hundreds 1-day old male broiler chicks (Cobb 500) were purchased from a commercial hatchery. The birds fed on the starter basal diet for 6 days. From day 7 of age onwards, the birds were distributed, at random, into 4 groups. Each group included 5 replicates with 10 chicks per replicate. The birds in group 1 were not administered lemongrass extract (control, LGX0) while chicks in group 2 (LGX100), 3 (LGX200) and 4 (LGX300) were administered the aqueous extract of lemongrass in drinking water at levels of 100, 200 and 300 ml/l, respectively. The experimental period lasted for 35 day. The chicks were housed in a floor pen, and wheat straw was used as a litter. An artificial lightning period of 23 h per day was provided throughout the experiment. The initial brooding temperature was 33 °C in the first week of age and reduced gradually 2 °C per week until reaching approximately 20 °C at the end of the experiment. Birds had a free access to water and feed along the experimental period. The diets were formulated to surpass the nutrient requirements of broilers according to the nutritional specifications guide of the breed (Table 1). The feeding period was divided into three phases, in which starter (0–14 day), grower (15–28 day) and finisher (28–35 day) diets were fed. All chicks were kept under similar managerial conditions. The vaccination program for all groups was also as follows: bivalent live infectious bronchitis and Newcastle vaccine, MA5+Clone 30 (Nobilis® Ma5-Clone30, MSD, Intetrvet Int., The Netherlands) at 5 days of age via eye drop (ED), bivalent inactivated avian influenza subtype H5 plus Newcastle vaccine (MEFLUVAC® H5+ND7, MEVAC, Egypt) at 10 days of age through subcutaneous route with a dose of 0.5 mL/bird, Gumboro intermediate plus, Bursine plus® (Zoetis, USA) at 12 days of age via ED and live Newcastle, laSota® (MSD, Intertvet Int., The Netherlands), at 18 days of age via ED.

**Table 1:** Nutritional composition (%) of the experimental broilersˈ diets (as fed).

Composition	Diet		
	Starter (0–14 d)	Grower (15–28 d)	Finisher (29–35 d)
Yellow corn	54.93	60.88	70.17
Soybean meal, (48 % CP)	38.40	33.30	25.46
Soy oil	2.60	2.90	1.50
Limestone	2.20	1.75	1.75
Di- calcium phosphate	0.85	0.20	0.20
Common salt	0.45	0.45	0.45
Vitamin premix^a^	0.10	0.10	0.10
Mineral premix^b^	0.20	0.20	0.20
DL-methionine, (98 %)	0.20	0.15	0.10
Mixed enzymes^c^	0.030	0.030	0.030
Phytase^d^	0.005	0.005	0.005
Coccidiostat^e^	0.020	0.020	0.020
Anti-mycotoxin^f^	0.016	0.016	0.016
Total	100	100	100
Calculated analysis			
ME, (kcal/kg)	3000	3100	3100
Crude protein	23	21	18
Fat	2.60	2.75	3.02
Fiber	2.49	2.45	2.41
Calcium	1.10	0.77	0.75
Available phosphorus	0.50	0.38	0.43
Total lysine	1.30	1.05	1.08
Methionine	0.57	0.45	0.47
Cysteine	0.33	0.28	0.28
Met + Cys	0.90	0.73	0.75
Arginine	1.33	1.07	1.00

a Vitamin premix provides per diet: Vit. A; 12000000 IU, Vit. E: 400000 mg, Vit. Bl: 2000 mg. Vit. B2: 160000 mg, Vit.B6: 5000 mg, Vit, B12: 12 mg, Niacin: 45000 mg, Pantothenic acid: 12000 mg, Vit. K: 3000 mg, Vit. D3; 3000000 IU, Biotin: 70 mg and Folic acid: 2000 mg.

b Trace mineral premix provides per diet: Choline: 3600000 mg, Copper: 10000 mg, Iodine: 1000 mg, Iron: 30000 mg, Manganese: 100000 mg, Zinc: 600000 mg, and selenium: 400 mg, cobalt: 100 mg.

c Combo® Enzyme Blend consists of: Cellulase 75,000 CU units/kg, Fungal amylase 30,000 SKB units/kg, Fungal protease 1,000,000 HUT units/kg, Neutral protease 100,000 PC units/kg, Alkaline protease 1.2 Anson units/kg, Xylanase 20,000 XU units/kg, Beta-glucanase 20,000 BG units/kg, Hemicellulase 20,000 HCU units/kg and Lipase 75,000 FIP units/kg.

d Axtra® PHY 10000 TPT, 6-phytase 10000 FTU/g.

e Diclazuril 500 mg, Atozuril® (ATco pharma).

f Mycofix® Select 3, feed additives that protect broiler health by deactivation of mycotoxin.

### 2.2 Growth performance and economic efficiency

Weight change and diet consumption were recorded weekly for 4 weeks. BWG and FCR, and EEI were calculated [23].

### 2.3 Hematological and biochemical biomarkers

At the end of the experiment ten blood samples were collected from each group in tubes without anticoagulant. The samples were allowed to clot for 1 h, at the room temperature and then centrifuged at 3000 r.p.m. for 20 min for serum separation. Collected sera were stored in a deep freezer at −20 °C until the chemical analyses. At the time of analysis, the samples were thawed and total protein, albumin, aspartate transferase (AST), alanine transferase (ALT), cholesterol, creatinine, urea, total antioxidant capacity (TAC), and MDA were detected using a spectrophotometer (SELECTA®UV-2005) and commercial kits (Bio-diagnostic, Egypt) following the manufacturer's instructions. Triiodothyronine (T3), thyroxine (T4) and corticosterone hormones (ng/ml) were tested using the radioimmunoassay technique by chemical kits. Moreover, Aliquot of blood was obtained to count the white blood cells (WBCs), red blood cells (RBCs), measure hemoglobin (Hb), packed cell volume (PCV), mean corpuscular volume (MCV), mean corpuscular hemoglobin (MCH), lymphocytes (L), heterophils (H), ratio of H/L, mean corpuscular hemoglobin concentration (MCHC) and platelets.

### 2.4 Gene expression

At the end of the experimental period, 20 birds (five per group) were sacrificed. The jejunum was rapidly separated, then opened, and the digesta was washed with physiological saline and then stored at −80 °C for further detection of mRNA gene expression. RNA extraction was done by TRIzol reagent (Invitrogen/Life Technologies, Carlsbad, CA, USA) and NanoDrop for quantification. The cDNA was synthesized from RNA (cDNA Reverse Transcription kits; Applied Bio systems), and then samples of cDNA were stored at −20 °C. Real-time polymerase chain reaction was applied to quantify expression of antioxidant biomarkers (Catalase, GPX1 and SOD1), and immune biomarkers (IL1, IL6, INF-ᵧ and TNF). One pair of primers for each gene was used based on the gene sequences found at GenBank (http://www.ncbi.nlm.nih.gov), and tow reference housekeeping gene, β actin and GAPDH, as found in (Table 2).

**Table 2:** Primers sequences used in relative gene expression quantification.

Target Genes	Primer sequence (5′–3′)		Amplicon (bp)	Accession number	References
Reference genes					
β-actin	Forward Reverse	AGCGAACGCCCCCAAAGTTCT AGCTGGGCTGTTGCCTTCACA	139	NM_205518.1	[24]
GAPDH	Forward Reverse	GCTGGCATTGCACTGAATGAC CACTCCTTGGATGCCATGT	113	NM_204305.1	[25]
Antioxidant related genes					
Catalase	Forward Reverse	ACTGGTGCTGGCAACCC ACGTGGCCCAACTGTCAT	57	NM_001031215.2	[26]
GPX-1	Forward Reverse	GCGACTTCCTGCAGCTCAACGA CGTTCTCCTGGTGCCCGAAT	99	NM_001277853.3	[27]
SOD-1	Forward Reverse	CGGGCCAGTAAAGGTTACTGGAA TGTTGTCTCCAAATTCATGCACATG	83	NM_205064.2	[26]
Immunity related genes					
IL-1ᵦ	Forward Reverse	TGCTTCGTGCTGGAGTCACCC GGCCGGTACAGCGCAATGTT	98	XM_015297469.1	[25]
IL6	Forward Reverse	AGCGAAAAGCAGAACGTCGAGTC GCCGAGTCTGGGATGACCACTTC	107	XM_015281283.2	[25]
IFN-ᵧ	Forward Reverse	AACAACCTTCCTGATGGCGTGA GCTTTGCGCTGGATTCTCAAGT	89	NM_205149.1	[25]
TNF	Forward Reverse	CCCCTACCCTGTCCCACAA TGAGTACTGCGGAGGGTTCAT	67	NM204267	[28]

Each reaction composed of 1.5 μl of 1 μg/μl cDNA, ten μl SYBR Green PCR Master Mix (Quanti Tect SYBR Green PCR Kit, Qiagen), one μM of each forward and reverse primer for each gene and nuclease-free water to a final volume of 20 μl. The reactions were then quantified on an Applied Biosystem Real-time PCR 7500 Fast detection system under the following conditions: 95 °C for 10 min (first denaturation and 40 cycles of 95 °C for 15 s (second denaturation stage) followed by 60 °C for 1 min (annealing and extension stage). Comparing the Ct method (2^−ΔΔCt^) of target genes to the house keeping genes was used to calculate the changes in gene expression comparative [29].

### 2.5 Cecal bacterial counts

At the end of the experimental period, ten birds per group were randomly selected and euthanized, and the ceca were sealed [30], and stored at −80 °C until counting the total number of both aerobic and anaerobic bacteria using the protocols which stated by Abdel-Latif et al. [31].

### 2.6 Histology

Morphological examination was done for intestine (about 3 cm jejunal samples; 1 cm before the midpoint). Five birds/group tissue samples were taken and were fixed in neutral buffered formalin (10 %) for 2–5 days. Samples were dehydrated in ascending grades of ethanol starting from 70 % to absolute one. Then using xylene (three changes) for clearance of the samples and the samples after that was put in melted paraffin in oven at 56 °C for (three changes). Finally cut using rotatory microtome the blocks of the processed samples. Thin paraffin sections (5-7 μm-thick) were cut from the samples’ blocks and mounted on coated glass slides and dried in an incubator for 30–60 min at 45 °C then stained with hematoxylin and eosin (H and E) for general inspection of the organ based on Bancroft and Layton [32]. Jejunum length, depth and crypt were measured by Image J software (NIH). The VH (villi height) and CD (crypt depth), measured by Image J software (NIH) and then divided the VH/CD to obtained ratio.

### 2.7 Experimental statistics

SPSS 20 was applied using Variance Analysis (ANOVA). Significant differences with Tukey's post hoc test were calculated at *p* < 0.05. RT-PCR data were analyzed using Graphpad prism 5.

## 3 Results

### 3.1 Performance and economic efficiency

The results of the growth performance and economic efficiency index of broiler chickens administered varying levels of aqueous lemon grass extract at drinking water is shown in Table 3 at 35 days of age. Lemongrass had a substantial impact on BW and BWG values (*p* ≤ 0.001). LGX200 had the highest BW by 9.51 %, followed by LGX100 which increased by 4.73 %. However, they were dropped in LGX300 by 9.27 %. In general, bird's treated with water enriched with 300 ml LGX/liter consumed less feed, besides recording a highest FCR and low EEI compared to other groups. In contrast, FI and EEI were similar in LGX200, LGX100 and control group.

**Table 3:** Growth performance and economic efficiency index of broilers chicks administered varying levels of aqueous lemongrass extract.

Items	Lemongrass extract supplementation groups				SEM^1^	*p-value*
	LGX0	LGX100	LGX200	LGX300		
IBW, g	142	142	143	143	0.42	0.815
FBW, g	1749^b^	1832^ab^	1916^a^	1587^c^	30.5	0.001
BWG, g/day	57.4^b^	60.4^ab^	63.3^a^	51.6^c^	1.09	0.001
FI, g/day	90.8^a^	89.1^a^	88.1^a^	83.9^b^	0.64	0.001
FCR	1.58^a^	1.48^b^	1.39^b^	1.63^a^	0.03	0.001
EEI (%)	98.7^a^	99.6^a^	100^a^	81^b^	0.69	0.01

Means with different superscripts within the same row are significantly different (*p* ≤ 0.05). IBW, initial body weight; FBW, final body weight; BWG, body weight gain; FI, feed intake; FCR, feed conversion ratio; EEI, Economic efficiency index = lowest feed cost per kilogram of weight gain among treatment/cost of treatment.

### 3.2 Hematological and biochemical biomarkers

Impact of lemongrass extract on hematological and biochemical biomarkers is presented in Table 4, Table 5. Significant differences of RBCs, Hb and PCV were existed for broilers treated with 200 and 300 ml LGX/L compared with those for control and 100 ml LGX/L. Also, it appears from data of Table 4 that all bird groups represented an increase (*p* ≤ 0.001) of Hb, PCV, MCH, and lymphocytes due to experimental LGX supplementation compared to control. No significant differences were observed between WBCs. Whereas, 100 and 200 ml LGX/L had the lowest (*p* ≤ 0.001) ratio of H/L.

**Table 4:** Blood biomarkers of broilers chicks administered varying levels of aqueous lemongrass extract.

Items	Lemongrass extract supplementation groups				S E M	*p*-value
	LGX0	LGX100	LGX200	LGX300		
RBC, 10^6^/mm^3^	2.35^b^	2.43^b^	2.63^a^	2.62^a^	0.03	0.001
Hb, g/dl	8.77^c^	9.67^b^	0.90^a^	10.93^a^	0.08	0.001
PCV, %	26.1^c^	29.5^b^	31.4^a^	31.9^a^	0.16	0.001
MCV	111^b^	121^a^	119^a^	122^a^	1.71	0.017
MCH, pg	37.4^c^	39.8^b^	41.5^ab^	41.7^a^	0.43	0.001
MCHC, g/dl	33.6^ab^	32.8^b^	34.7^a^	34.2^a^	0.37	0.042
WBC, 10^3^/mm^3^	13.1	13.4	14.2	13.8	0.35	0.304
Lymphocyte, %	60.4^c^	65.5^a^	65.9^a^	63.4^b^	0.50	0.001
Heterophils, %	31.7	29.2	28.8	30.9	0.80	0.074
H/L ratio	0.52^a^	0.44^c^	0.43^c^	0.48^b^	0.01	0.001
Platelets, 10^3^/ml	117^c^	131^bc^	144^b^	180^a^	6.20	0.001

Means with different superscripts within the same row are significantly different (*p* ≤ 0.05). Hb: hemoglobin concentration, RBC: red blood cell count, PCV: packed cell volume, MCV: mean corpuscular volume, MCH: mean corpuscle hemoglobin, MCHC: mean corpuscle hemoglobin concentration, WBC: white blood cell count, L lymphocytes cell, H: heterophile cells. Means with different superscripts within the same row are significantly different (p ≤ 0.05).

**Table 5:** Biochemical biomarkers of broilers chicks administered varying levels of aqueous lemongrass extract.

Items	Lemongrass extract supplementation groups				S E M	*p*-value
	LGX0	LGX100	LGX200	LGX300		
Total protein, g/dl	5.17^b^	5.33^a^	5.40^a^	5.10^b^	0.04	0.001
Albumin, g/dl	2.43^b^	2.63^b^	3.23^a^	2.46^b^	0.102	0.01
Globulin, g/dl	2.73	2.69	2.16	2.63	0.154	0.15
AST, u/l	164^a^	156^ab^	144^c^	151^bc^	3.21	0.01
ALT, u/l	7.15	6.85	6.90	5.85	0.45	0.29
Cholesterol, mg/dl	145^a^	130^b^	129^b^	128^b^	3.51	0.001
Creatinine, mg/dl	0.29^a^	0.23^b^	0.22^b^	0.21^b^	0.009	0.001
Urea, mg/dl	10.90	10.63	11.06	11.10	0.192	0.46
TAC, mM/L	2.05^b^	2.49^a^	2.62^a^	2.56^a^	0.109	0.01
MDA, nm/L	17.9^a^	11.9^b^	13.2^b^	12.9^b^	0.856	0.01
Cortecosterone, nm/L	9.63^b^	10.50^b^	7.43^c^	12.72^a^	0.523	0.001
T3, ng/Ml	2.08^b^	2.74^a^	2.82^a^	2.10^b^	0.044	0.001
T4, ng/mL	4.61	4.49	4.47	4.45	0.088	0.63
T3/T4 ratio	0.450^b^	0.611^a^	0.631^a^	0.470^b^	0.084	0.001

AST: aspartate aminotransferase, ALT: alanine aminotransferase, ALP: alkaline phosphatase, TAC: total antioxidants capacity, MDA: malondialdehyde, T3: thyroid hormone, T4: thyroxine hormone. Means with different superscripts within the same row are significantly different (*p* ≤ 0.05).

Birds of LGX100 and LGX200 had increased (*p* ≤ 0.01) total protein and albumin compared to control and LGX300, however, the globulin was similar among the experimental groups. LGX groups decreased (*p* ≤ 0.001) serum cholesterol level by 10.12 %, 11.03 % and 11.45 % with different doses, respectively, compared with control. Serum AST level was decreased (*p* ≤ 0.01) for broilers treated with LGX200 and LGX300 compared with control group. LGX groups had low creatinine (*p* ≤ 0.001) compared control without any statistical differences for urea concentration. Total antioxidant capacity (TAC) was high (*p* ≤ 0.01) in all supplemented groups. The reverse was found for serum malondialdehyde (MDA). Broilers of LGX200 had a lowest (*p* ≤ 0.001) level of corticosterone hormone. T3 hormone was higher (*p* ≤ 0.001) in LGX100 and LGX200 than the others.

### 3.3 Gene expression

**Fig. 1:**
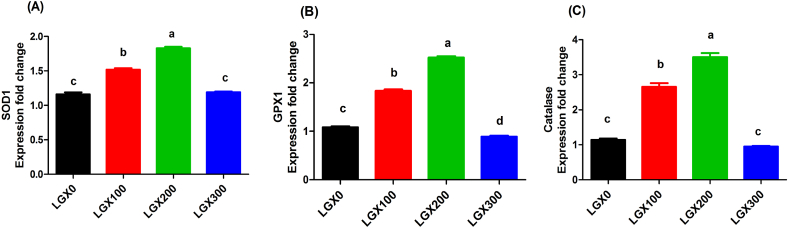
RT-PCR validation of the antioxidant genes SOD1; superoxide dismutase **(A)**, GPX1; glutathione peroxidase **(B)**, and Catalase **(C)** of birds with aqueous lemongrass extract. Columns (control (LGX0), 100 ml lemongrass/1 L drinking water (LGX100), 200 ml lemongrass/1 L drinking water (LGX200) and 300 ml lemongrass/1 L drinking water (LGX300) carrying different letters (a, b, c and d) using Tukey's post hoc test are significantly different at *p* < 0.05.

**Fig. 2:**
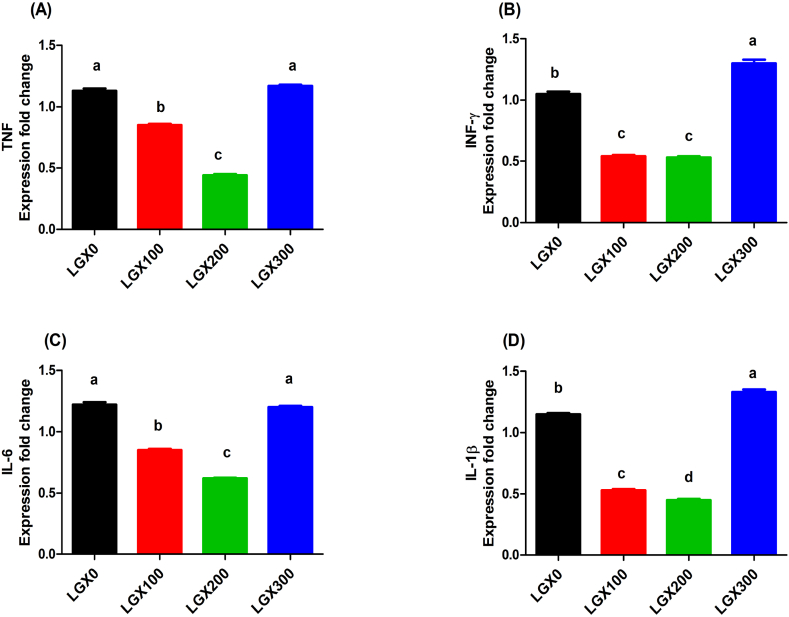
RT-PCR validation of the immune related genes; tumor necrotic factor (TNF) **(A)**, interferon gamma (IFN-ᵧ) **(B)**, interleukin 6 (IL-6) **(C)**, and interleukin 1 beta (IL-1ᵦ) **(D)** of birds with aqueous lemongrass extract. Columns (control (LGX0), 100 ml lemongrass/1 L drinking water (LGX100), 200 ml lemongrass/1 L drinking water (LGX200) and 300 ml lemongrass/1 L drinking water (LGX300) carrying different letters (a, b, c and d) using Tukey's post hoc test are significantly different at *p* < 0.05.

Antioxidant genes expression in jejunum is shown in Fig. 1 (A–C). Up regulation (*p* < 0.001) of the expressions of superoxide dismutase 1 (SOD1), glutathione peroxidase 1 (GPX1), and catalase were in LGX200 group compared to other groups. While, the proinflammatory genes expression (tumor necrosis factor (TNF), interferon gamma (IFNᵧ) interleukin 6 (IL6), and interleukin 1 beta (IL1B)) showed down regulation (*p* < 0.001) in the LGX200 compared to others as shown in Fig. 2 (A–D).

### 3.4 Cecal bacterial count

All birds groups represented a decrease (*p* < 0.001) of caecum *E.coli* counts due to the experimental LGX supplementation compared to control group (Table 6). Inverse was true for Lactobacillus counts. The statistical analysis showed that LGX100 and LGX200 groups had the lower (*p* < 0.001) Salmonella counts than others. LGX200 was the best to decline intestinal pathogens.

**Table 6:** Cecal bacteriology of broilers chicks administered varying levels of aqueous lemongrass extract.

Items	Lemongrass extract supplementation					
	LGX0	LGX100	LGX200	LGX300	S E M^1^	*p-value*
*Escherichia coli*	5.77^a^	5.48^b^	5.43^b^	5.17^c^	0.05	0.001
Salmonella spp.	2.24^a^	2.25^a^	2.10^b^	1.97^c^	0.03	0.001
Lactobacillus spp.	6.48^c^	6.58^bc^	6.69^b^	7.08^a^	0.06	0.001

Means with different superscripts within the same row are significantly different (p ≤ 0.05).

### 3.5 Histology

**Fig. 3:**
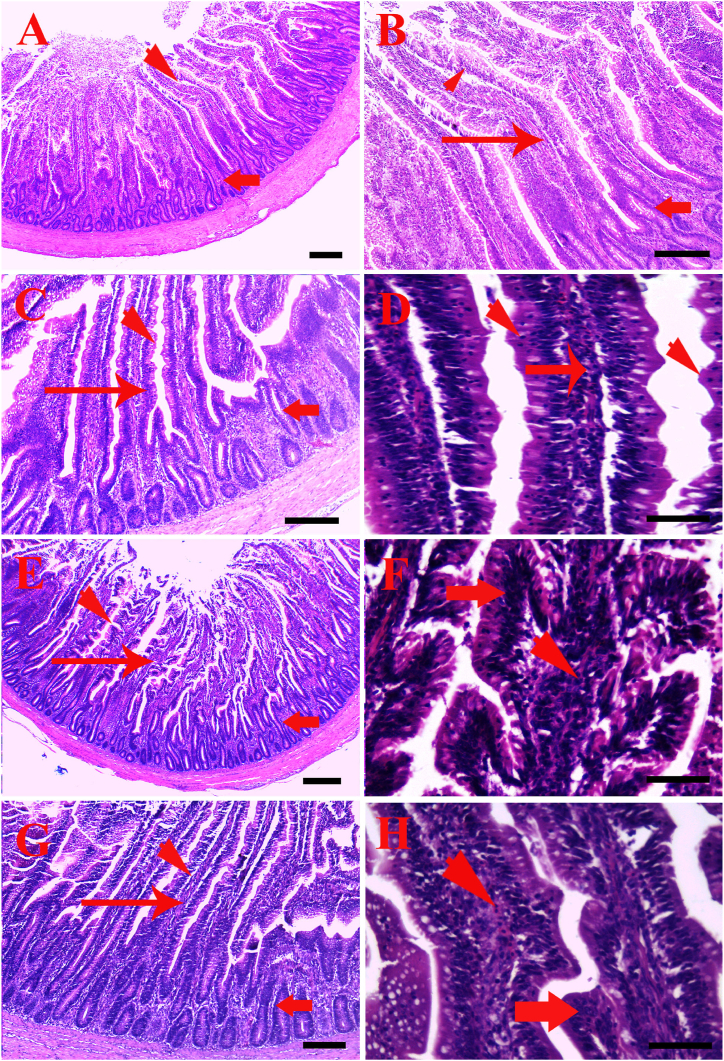
Photomicrograph of broiler jejunum treated by different doses of lemongrass and stained by hematoxylin and eosin. A, control group showing normal intestinal villi with lining epithelium (arrowhead) and intestinal crypt (arrow), scale bar = 200 μm. B, showing control group with normal lining epithelium (arrowhead), lamina propria (thin arrow) and intestinal crypts (thick arrow), scale bar = 100 μm. C, group treated by 100 ml/L (LGX100), lemongrass showing improved, protrude lining epithelium (arrowhead), lamina propria with lymphocytes infiltration (thin arrow) and intestinal crypts (thick arrow), scale bar = 100 μm. D, group treated by 100 ml/L (LGX100), lemongrass showing increase the villi length with proliferation of lining epithelium (arrowheads) and lamina propria with lymphocytes infiltration (thin arrow), scale bar = 50 μm. E, group treated by 200 ml/L (LGX200), lemongrass showing increase the length of villi with lining epithelium (arrowhead), lamina propria (thin arrow) and intestinal crypts (thick arrow), scale bar = 200 μm. F, higher magnification of villi of group treated by 200 ml/L (LGX200), lemongrass showing increased, higher proliferation of lining epithelium (arrowhead) and lamina propria with more lymphocytes infiltration (thin arrow), scale bar = 50 μm. G, group treated by 300 ml/L (LGX300), lemongrass showing improved villi length (arrowhead), lamina propria (thin arrow) and intestinal crypts (thick arrow), scale bar = 100 μm. H, higher magnification of villi epithelium of group treated by 300 ml/L (LGX300), lemongrass showing increased, limited bending of villi epithelium (arrowhead) and lamina propria width with higher lymphocytes infiltration (thin arrow), scale bar = 50 μm.

Jejunal histomorphology of birds with aqueous lemongrass extract is shown in (Table 7 and Fig. 3). Villus height and crypt depth were enhanced (*p* ≤ 0.001) in LGX200 and LGX100 compared to other groups. The normal finger-like projections of jejunal villi which extend into the intestinal lumen with few goblet cells located in between. Below the epithelium, lamina propria is a loose and had irregular cells of connective tissue. Most of the cells within the propria meshes of the collagen fibrils are plasma cells, although many other cell types can be found. The intestinal crypts located between the villi and extended deep in tunica mucosa. The tunica muscularis layers formed from smooth muscle and contain two layers the inner circular and outer longitudinal layer (Fig. 3A and B).

**Table 7:** Intestinal histology of broilers chicks administered varying levels of aqueous lemongrass extract.

Items	Lemongrass extract supplementation				*p-value*
	LGX0	LGX100	LGX200	LGX300	
Villus height (VH), μm	847.14 ± 40.39^d^	1467.05 ± 44.72^b^	1730.38 ± 15.56^a^	1035.45 ± 17.83^c^	0.001
Crypt depth (CD), μm	369.92 ± 24.78^b^	623.67 ± 21.49^a^	681.25 ± 21.11^a^	404.15 ± 21.25^b^	0.001
VH/CD	2.33 ± 0.20	2.35 ± 0.11	2.54 ± 0.34	2.59 ± 0.13	0.12

Means with different superscripts within the same row are significantly (p ≤ 0.05) different. VH, CD and VH/CD analysis were done for 5 replicates (n = 10).

Lemongrass was shown to increase the length of villi gradually by using different level of dose. In group supplied by 100 ml/L (LGX100), of lemongrass on drinking water showing improved, little protrude of lining epithelium, increased villi length, lamina propria with lymphocytes infiltration and intestinal crypts development (Fig. 3C and D). With increasing the dose of lemongrass 200 ml/L (LGX200), supplied group showing more, higher villi length, proliferation of lining epithelium for increasing absorption content and the width of villi was increase by more proliferation of connective cells and blood cells, crypts are improved with proliferated lining epithelium (Fig. 3E and F). When the dose of lemongrass increased 300 ml/L (LGX300) of treated group, showing moderate increase of villi length and the bending of lining epithelium decrease with little improvement in lamina propria with proliferation of cells and blood, intestinal crypts showing similar to previous group (Fig. 3G and H).

## 4 Discussion

Lemongrass had various phytochemical components such as saponins, phenols, resins, alkaloids, tannins, flavonoids, glycosides and terpenes, minerals as well as vitamin C which had various pharmacological actions. The poly phenolic, alkaloids, terpenoids, carotenoids, and some vitamins compounds which have antioxidant activity, as they work to protect the cells of the bird from oxidative stress by neutralizing and curbing harmful free radicals. These compounds have also a biological efficacy that allows them to act as antibiotics against bacteria and fungi [33]. Furthermore, many studies have illustrated that other compounds found in lemongrass, such as flavonoids and tannins which improve the performance and the health of the bird's digestive system [11]. Addition of LGX at level 200 ml/l and 100 ml/l in broiler drinking water had a beneficial influence on growth indices due to presence of flavonoids and tannins in LGX which improve the bird's appetite and health of digestive system [11,34]. Moreover, LGX can stimulate the production of digestive juice for efficient nutrients utilization and an increase in the rate at which digesta passes through the intestines [35]. Also, LGX contains poly phenolic, alkaloids, terpenoids, carotenoids, and some vitamins compounds which have a beneficial effect on bird health and performance due to its antioxidant and antimicrobial activity [13]. On the other hand, the improvements in gut health can enhance the digestion and absorption of nutrients [36] and boost feed conversion ratio [9,13].

The improvement of hematological parameters of broilers treated with LGX100 and LGX200 compared with control could be related to the fact that lemongrass is rich in iron and copper which enter into the process of forming erythrocytes and thus reduced anemia in broilers [37]. The antioxidant activity of flavonoids in herbs improved the hematological biomarkers which protect against any stress (PCV and heterophile/lymphocyte ratio), and fight against infections (WBCs count) [38,39]. Similarly, lemongrass extracts are efficacious in enhancing serum protein and albumin levels without any effect on globulin and this probably due to high phenolics and flavonoids contents [21,40]. On the other hand, the reduction of serum protein concentration for LGX300 may due to the reverse effect of ascorbic acid overdose (physiological stress) which lower the biological value of protein [41].

Lemongrass phenolic components which have antioxidant [42], cytoprotective properties and antihypertensive components has significant hypocholesterolemic [43], low levels of AST and ALT which stimulate the integrity of the liver and muscles [9]. Also, improvement of renal function biomarkers (urea and creatinine) and inhibited lipid peroxidation (low MDA and high TAC) as stated by Ojo et al. [44] were reported. One of the surprising results is that LGX200 could reduce serum corticosterone hormone concentration and thereby could ameliorate the anti oxidative stress or protect against stressors in birds, further research is needed. As a result, it has antihypertensive effect in animals due to stimulation of parasympathetic activity (Silva and Barbara, 2022). High corticosterone concentration of LGX300 could be related to overdose of citric acid in lemongrass which may increase the acidity of water as stated by Oviedo [45]. Thyroid hormones are involved in the regulation of metabolic pathways of all nutrients [46]. Increased T3 hormone was reported and metabolic cycle [21].

The antioxidant activity of lemongrass enhanced antioxidant gene expression [47]. The findings were in line with Li et al. [48] who recorded that an enhance in the activity of antioxidant enzymes (SOD and GPX) due to the supplementation of herbs in chicken. Similar results recorded by Alagawany et al. [13] who recorded that, total antioxidant capacity, GSH, and catalase were significantly increased by lemongrass inclusion compared with the control. The supplementation lemongrass enhanced the immunity and resistance against diseases [49]. Under normal settings, pro-inflammatory cytokines (IL1B, IL6 and TNF) are not released but are secreted in response to immune system stimulation [50], so, downregulation of pro-inflammatory cytokines expression means that the birds are under normal condition.

Lemongrass enhanced the gut health by modifying the microbial counts and intestinal morphology, stimulating immunity and antioxidants, improving the production of the broilers [51] and quails [9,13]. The antioxidant activity was improved by LGX may be due to superoxide, and LGX scavenges hydroxyl radicals; metal ions chelation and the formation of inactive complexes; upregulation the expression of the antioxidant genes as the results shown (SOD1, GPX1 and catalase), and stimulation of endogenous antioxidant enzyme production in cells [52]. Lemongrass modulatory effect on indigenous intestinal flora is thought to be advantageous in developing the gut's immune system [53]. The antibacterial activity of natural extracts is intimately linked to the presence of phenolic and polyphenolic chemicals, which operate as potent active molecules with significant antioxidant and antimicrobial properties [17,54]. Animal health is directly related to the balance of intestinal microbiota; both good and detrimental microorganisms naturally inhabit the gastrointestinal tract of poultry [9]. Vitamin synthesis, gas emission, digestion, and nutrient absorption are all aided by beneficial microbes, restricting the growth of dangerous bacteria [55]. Lemongrass contains two main components citral and pinene which possesses a broad spectrum of antibacterial activities [56].

Histomorphological examination which was unprecedented before shows normal small intestine (jejunum) of broiler involved in the absorption of the bulk of nutrients. Lemongrass increased the length of villi gradually by increasing the dose; improved and little protrusion of lining epithelium and increased villi length (LGX100), while higher villi length and proliferation of lining epithelium for increasing absorption content (LGX200). However, moderate increase in villi length and the bending of lining epithelium decreased (LGX300). These findings are in accordance of Lan et al. [57] who noted that organic acids and essential oils as types of prebiotics may not only benefit for the intestinal microbiome but also progress the intestinal epithelial cells integrity, which further increases the nutrients absorption and promote the growth performance of animals. The improved gut morphology of LGX200 group confirmed the enhanced nutrient absorption, thus improving and preserving the microstructure of the intestine, thus improving growth performance. Positive changes in the gut structure of chickens funded with symbiotic and probiotics in terms of more villus width and higher villus surface area in the ileum and jejunum parts and deeper crypts [58], thus confirming the beneficial impacts on intestinal morphometric characteristics.

## 5 Conclusion

The results indicate that using of lemongrass aqueous extract at levels 100 and 200 ml/l in broilersˈ drinking water can enhance the growth performance, blood parameters and gut health status. LGX at a 200 ml/l supplementation level achieved the best results followed by a 100 ml/l level. So, it can be recommended to use LGX200 ml/l in broiler drinking water. Moreover, LGX increased the height and width of intestinal villi, resulting in high absorption of dietary nutrients. Supplementation of LGX to broiler drinking water stimulated the immunity of poultry through increasing the level of immunological blood indicators as well as the local intestinal immunity.

## Ethics statement

All animal procedures were conducted in accordance with the standards set forth in the guidelines for the care and use of experimental animals. The study protocol was approved by the Animal Ethics Committee at Faculty of Veterinary Medicine, Damanhour University (DMU/VetMed-2023/011).

## Data availability statement

The authors confirm that the data supporting the findings of this study are available within the article. Then, the manuscript is available to the readers after acceptance according to the policy of the journal without any objection.

## CRediT authorship contribution statement

**Amany A. El-Sahn:** Writing – review & editing, Validation, Methodology, Data curation. **Eman A. Manaa:** Writing – review & editing, Methodology, Formal analysis, Data curation. **Amal M. EL-Barbary:** Writing – original draft, Validation, Software, Methodology. **Ayman M. Khalifah:** Writing – original draft, Software, Methodology, Investigation, Data curation. **Sahar Fayez:** Writing – original draft, Methodology, Formal analysis. **Asmaa S.A. Abdel-Daim:** Writing – original draft, Software, Methodology, Investigation. **Ghadeer M. Albadrani:** Writing – review & editing, Resources, Funding acquisition. **Mohamed M. Abdel-Daim:** Writing – review & editing, Supervision, Conceptualization. **Mervat A. Abdel-Latif:** Writing – review & editing, Supervision, Project administration, Conceptualization.

## Declaration of competing interest

All authors have no competing financial or non financial interests that could influence the work in this manuscript.
